# Role of Anticonvulsants in the Management of Posttraumatic Epilepsy

**DOI:** 10.3389/fneur.2016.00032

**Published:** 2016-03-22

**Authors:** Batool F. Kirmani, Diana Mungall Robinson, Ekokobe Fonkem, Kevin Graf, Jason H. Huang

**Affiliations:** ^1^Epilepsy Center, Department of Neurology, Baylor Scott & White Health Neuroscience Institute, Texas A&M Health Science Center College of Medicine, Temple, TX, USA; ^2^Department of Psychiatry, University of Virginia Medical Center, Charlottesville, VA, USA; ^3^Division of Neuro-oncology, Department of Neurosurgery, Baylor Scott & White Health Neuroscience Institute, Texas A&M Health Science Center College of Medicine, Temple, TX, USA; ^4^Department of Neurosurgery, Baylor Scott & White Health Neuroscience Institute, Texas A&M Health Science Center College of Medicine, Temple, TX, USA

**Keywords:** posttraumatic seizures, traumatic brain injury, epilepsy, anticonvulsants, management

## Abstract

Posttraumatic seizures (PTS) have been recognized as a major complication of traumatic brain injury (TBI). The annual incidence of TBI in the United States is 1.7 million. The role of anticonvulsants in the treatment of posttraumatic epilepsy (PTE) remains uncertain. Based on current studies, however, anticonvulsants have been shown to reduce early PTS occurring within the first 7 days, but little to no benefits have been shown in late PTS occurring after 7 days. In this paper, we provide a mini review of the role of anticonvulsants and current advances in the management of PTE.

## Posttraumatic Epilepsy

Posttraumatic epilepsy (PTE) due to traumatic brain injury (TBI) has many causes, including wartime combat, vehicle accidents, work-related injuries, and sports injuries. Wartime combat injuries, especially blast injuries and penetrating head injuries (PTI), have shown to increase the risk of seizures, as that of blast models of TBI (1–4). The annual incidence of TBI is estimated to be 1.7 million in the United States, and seizures have been recognized as one of the major complications of this condition (5). The incidence of PTE was described by Annegers and colleagues who conducted a retrospective study in order to identify the characteristics of brain injuries that are associated with the development of seizures for 50 years. The results showed that the severity of the injury was correlated with the interval during which the risk of seizures was increased, even after more than 20 years post injury (6). The other study of interest was the Vietnam Head Injury Study (VHIS) that was a prospective, longitudinal follow-up of 1,221 Vietnam War Veterans who had PTI. The prevalence of PTE in this cohort was 45–53%. Patients with PTI carry a high risk of PTE even for decades; so, long-term medical follow-up is required (7). Similarly, the prospective study by Salazar and colleagues showed that seizure frequency in the first year predicted future severity of seizures. A higher seizure frequency was seen in the first year and was also associated with subjects having a longer duration of epilepsy and persistent seizures (8).

## Role of Anticonvulsants in the Management of Posttraumatic Epilepsy

The seizures after head injury result in secondary brain damage, which involves increased intracranial pressure, increased metabolic brain demands post head injury, and excessive release of neurotransmitters, which result in further complicating the existing damage. The main goal of anticonvulsants is to minimize the brain damage by preventing early seizures (9).

The other role of anticonvulsants apart from antiseizure activity is the neuroprotective effect, which has been demonstrated in animal models. Phenytoin, which is still considered as an agent of choice, has been shown to have neuroprotective properties in animal models. Vartanian and colleagues showed that phenytoin has been linked with decreased neuronal damage in neonatal rats following hypoxia (10). Another study by Tasker and colleagues showed similar results in rat hippocampal structures (11). Researchers suggested that neuroprotective effects were related to a blockage of voltage-dependent sodium channels during hypoxia, which decreased the spread of calcium-induced neurotoxicity following hypoxic brain injury (10, 11).

Posttraumatic seizures (PTS) are divided into two subgroups, early and late PTS. Early seizures occur within the first 7 days after brain injury, and late seizures occur after 7 days of injury. These definitions are important in terms of management and predicting prognosis of PTE (12).

The prospective randomized trials did not show promising results of the role anticonvulsants in the management of PTS. The randomized clinical trials are summarized in Table 1. No significant differences were seen in the treatment versus the non-treatment groups (13–20). Summary of selected non-randomized trials for posttraumatic seizure prevention was shown in Table 2, which also did not show a significant difference between groups (21–28).

**Table 1:** **Summary of selected randomized controlled trials (RCT) for posttraumatic seizure prevention**.

Reference	Study design	Number of patients randomized (*N*)	Methods	Outcome
Dikmen et al. (13)	RCT	124	Phenytoin versus placebo Patients were randomized to receive either PHT or placebo for 1 year and observed one more year without medication	No significant differences seen in neuropsychological examinations in 1 year between the 2 groups
Temkin et al. (14)	RCT	123	Phenytoin versus placebo Treatment was started within 24 h of injury for 1 year and then 2 groups were followed for 2 years	Early seizures: improvement seen in the PHT GROUP Late seizures: no difference between the 2 groups
Young et al. (15)	RCT	244	Phenytoin versus placebo Treatment was started within 24 h of injury	Early seizures: no difference between the 2 groups Late seizures: study was not designed to determine late seizure outcome
Young et al. (16)	RCT	179	Phenytoin versus placebo Treatment was started within 24 h of injury and 2 groups were followed for 18 months to determine late seizure outcome	Early seizures: study was not designed to determine early seizure outcome Late seizures: no difference between the 2 groups
McQueen et al. (17)	RCT	164	Phenytoin versus placebo Two groups were followed for 2 years Occurrence of seizures was used as outcome measure	Early seizures: study was not designed to determine early seizure outcome Late seizures: no difference between the 2 groups
Szaflarski et al. (18)	RCT	52	Phenytoin versus levetiracetam Treatment was started within 24 h of injury between the 2 groups	Early seizures: no difference between the 2 groups Late seizures: study was not designed to determine late seizure outcome
Temkin et al. (19)	RCT	379	Phenytoin for 1 week versus valproate for 1 month versus valproate for 6 months Treatment was started within 24 h of injury Follow-up of these groups continued for 2 years	Early seizures: no difference among 3 groups Early seizures: no difference among 3 groups
Manaka (20)	RCT	191	Phenobarbital versus no treatment Treatment was started within 4 weeks post head injury They received full dose for 2 years and tapered off in third year Follow-up in 5 years	Early seizures: study was not designed to determine early seizure outcome Late seizures: no difference among 3 groups

**Table 2:** **Summary of selected non-randomized trials for posttraumatic seizure prevention**.

Reference	Study design	Number of patients randomized (*N*)	Methods	Outcome
Servit and Musil (21)	Non-RCT	167	Treatment group (*n* = 143) were administered phenytoin or phenobarbital Control group (*n* = 24) where conventional treatment was used Duration: 2 years	Early seizures: not applicable Late seizures: 25% in the control group and 2.1% in the treatment group
Inaba et al. (22)	Prospective controlled trial	813	Participants were administered either levetiracetam or phenytoin for 7 days	Early seizures: no difference between the 2 groups Late seizures: not applicable
Kruer et al. (23)	Retrospective cohort	109	Retrospective review of patients who received levetiracetam or phenytoin	Early seizures: no difference between the 2 groups Late seizures: not applicable
Gabriel and Rowe (24)	Cohort	19	Participants were divided based on levetiracetam and phenytoin prophylaxis Follow-up interview conducted to assess seizure outcome	Early seizures: no difference between the 2 groups Late seizures: no difference between the 2 groups
Jones et al. (25)	Cohort	27	Phenytoin versus levetiracetam administered during first 24 h post severe TBI	Early seizures: no difference between the 2 groups Late seizures: not applicable
Bhullar et al. (26)	Case-control	93	Phenytoin versus no treatment to determine occurrence of early seizures	Early seizures: no difference between the 2 groups Late seizures: not applicable
Formisano et al. (27)	Retrospective and prospective	137	Anticonvulsants versus no treatment Study 1: prospective Study 2: retrospective	Study 1 – No difference between the 2 groups Study 2 – Late seizures higher in the treated group
Watson et al. (28)	Cohort	404	Glucocorticoids administered within 1 day versus no glucocorticoids	Early seizures: not applicable Late seizures: no difference between the 2 groups

Temkin and colleagues showed that phenytoin was considered effective in preventing provoked seizures and promising at preventing unprovoked seizures. Carbamazepine was considered effective in preventing provoked seizures after TBI, although its status was considered uncertain in preventing unprovoked seizures. Phenobarbital was considered promising at preventing provoked seizures and uncertain at preventing unprovoked seizures. Finally, the combination of phenytoin and phenobarbital was considered promising to prevent provoked and unprovoked seizures. It was also shown that provoked seizures showed promising results, but for unprovoked seizures, no drugs were shown to be effective. AEDs prescribed to prevent epileptogenesis should be avoided until clinical trials have found a drug for this purpose (29). Similarly, Chang and Lowenstein conducted a literature review of the evidence of AED prophylaxis in patients with severe TBI in order to guide better practice recommendations. Patients given phenytoin prophylaxis compared to controls had a significantly lower risk of early PTS in pooled class I studies. There were no significant differences in the risk of late PTS patients receiving phenytoin, carbamazepine, or valproate prophylaxis versus controls in pooled class I and class II studies. In these studies, adverse effects were frequent, but mild and serum AED levels were suboptimal. The authors concluded that phenytoin prophylaxis is effective in decreasing the risk of early PTS in adult patients with severe TBI. However, late PTS are not decreased by AED prophylaxis (30). Current guidelines issued by the Brain Trauma Foundation and the American Academy of Neurology (AAN) for the management of severe TBI recommend seizure prophylaxis only for 7 days post injury. Phenytoin still remains the desired treatment because it has been extensively studied and there is proven evidence of its efficacy. The other antiepileptic agents, such as phenobarbital, valproate, and carbamazepine, have gone through limited trials as compared to phenytoin and their adverse effect profiles and pharmacodynamics properties still make phenytoin the desired antiepileptic for early prophylaxis of PTS (30).

The new anticonvulsants were favored over the older agents because of their unique pharmacokinetic properties, fewer serious side effects, and fewer drug–drug interactions (31). A second generation anticonvulsant that has generated particular interest is levetiracetam (23). Jones and colleagues conducted a retrospective study, which showed that levetiracetam can be used as an alternative to phenytoin, but the study was limited due to a small sample size (25). Szaflarski and colleagues conducted the first prospective randomized comparative trial of intravenous levetiracetam versus phenytoin for seizure prophylaxis in the neurointensive care unit setting, which showed that levetiracetam can be used as an alternative to phenytoin for seizure prophylaxis in the neuroscience intensive care setting. This study had limitations, including a small sample size and a lack of data reported on the concomitant use of sedating agents for induction of pharmacologically-induced coma (18). Kirmani and colleagues also conducted a literature review on the role of intravenous levetiracetam in seizure prophylaxis of severe TBI patients, which showed that levetiracetam can be used as an option in acute PTS (32). Gabapentin (GBP) is another anticonvulsant that acts at the α2δ-1 submit of the L-type calcium channel. It has been shown that chronic administration of GBP after cortical injury is antiepileptogenic in the undercut model of PTE. The results suggest that it may have a neuroprotective effect and may also decrease excitatory synapse formation. These results suggest the potential use of GBP as an anticonvulsant following TBI (33). A meta-analysis by Zafar and colleagues also concluded that there is no particular drug that is superior in preventing early seizures (34).

Wroblewski and Joseph reported 10 case studies of TBI patients treated with intramuscular midazolam for acute seizure cessation after other benzodiazepine drugs had failed. Slight to moderate sedation was the only reported side effect. The study was limited due to small sample size and was conducted to treat rather than prevent PTS (35).

The role of anticonvulsants in early PTS seems favorable as compared to late PTS. Anticonvulsants are found to be effective in patients who develop PTE.

Phenytoin remains the most commonly used anticonvulsants, but the side effects do favor the use of newer anticonvulsants, e.g., levetiracetam because of lack of drug–drug interactions and availability in parenteral form. The cognitive side effects and non linear kinetics limit the use in certain patient populations (13). Carbamazepine has shown to be effective but drug–drug interactions and unavailability in parenteral form limits the use of this agent. Neurocognitive side effects were also seen in other older anticonvulsants, including Phenobarbital, which may mask the mental status findings in TBI patients because of the sedating effects. Valproate can cause coagulopathy which may result in intracranial hemorrhage (30, 31).

Unfortunately, limited scientific data exist, which are specific to PTE with other anticonvulsants, and there is a need for additional controlled randomized clinical trials to explore more options.

## New Directions in the Management of Posttraumatic Epilepsy

The PTE can be differentiated from PTS that are sequelae from TBI. The term PTE signifies recurrent seizure disorder due to TBI or any surgery on the brain (36).

Posttraumatic epilepsy remains a challenge despite new medications that have come in the last decade. We still face problems with effective seizure control. TBI is the most common cause of acquired focal epilepsy (37–40). The model that was commonly used to assess antiepileptogenic interventions is the rostral parasagittal fluid percussion injury model (rpFPI) (41–43). This model helps in mimicking closed head injury by reproducing destructive processes as well as regenerative inflammatory processes (44). This is now considered as an excellent model for PTE (41–44). This model may progress to intractable multifocal epilepsy after a few months post injury (45). Interestingly, this model has shown to represent a severe form of PTE which is not controlled by carbamazepine, valproic acid, and carisbamate (41, 42). One study using this model showed mild cooling of epileptogenic focus and prevention of recurrent seizures. Based on the above studies, prolonged and mild cooling has been tried in these subgroups of patients and was found to be safe and improve functional recovery (46–49).

The animal model data also show that it is possible to target anti-inflammatory agents that are used for other indications as alternative to anticonvulsants. Progesterone has been shown to have promising effects in several brain injury models (50). The smaller sample size in humans did show some positive results (51, 52). However, the current evidence is insufficient to support the use of progesterone in the management of TBI (53).

## Conclusion

Anticonvulsants have proven to be beneficial in the first 7 days post injury. Phenytoin still remains the anticonvulsant of choice because it is widely studied and researched as compared to other anticonvulsants. Levetiracetam seems to be a viable alternative because of its unique pharmacodynamics properties; however, more head-on prospective clinical trials are needed regarding phenytoin in order to prove its efficacy as a first line agent in PTE. Clinical trials are needed to study the efficacy of second and third generation anticonvulsants in the treatment of PTE. Clinical trials are also needed to prove the role of mild selective cooling in patients with PTE.

## Author Contributions

All authors listed, have made substantial, direct, and intellectual contribution to the work and approved it for publication.

## Conflict of Interest Statement

The authors declare that the research was conducted in the absence of any commercial or financial relationships that could be construed as a potential conflict of interest.
